# Palliative care in Ethiopia’s rural and regional health care settings: a qualitative study of enabling factors and implementation challenges

**DOI:** 10.1186/s12904-023-01283-5

**Published:** 2023-10-17

**Authors:** Atsede Aregay, Margaret O’Connor, Jill Stow, Nicola Ayers, Susan Lee

**Affiliations:** 1 Health and Nursing Sciences, University of Agder, Kristiansand, Norway; 2 School of Nursing, Mekelle University, Tigray, Ethiopia; 3 Nursing and Midwifery, Monash University, Melbourne, Australia; 4 Melbourne City Mission Palliative Care, Melbourne, Australia; 5 St Vincent’s Private Hospital, Melbourne, Australia; 6 Nurse Lecturer, School of Nursing, BPP University, London, UK

**Keywords:** Palliative care, Rural population, Ethiopia, Implementation, Qualitative research

## Abstract

**Background:**

Palliative care is limited in Ethiopia, particularly in rural areas, where more than 78% of the population live. Current initiatives and research are focused on urban settings and are primarily donor dependent. This study aims to explore the status of palliative care, enabling factors and implementation challenges in Ethiopia’s rural and regional health care settings.

**Methods:**

A qualitative regional case study was conducted with health professionals drawn from different health care settings, academic institutions and included health planners and practitioners. Focus groups were conducted with rural community members and face- to face- individual interviews were conducted with health professionals working in numerous roles as well as academic leaders.

**Results:**

Participants indicated that despite a few leaders being aware of the inclusion of palliative care in the Ethiopia national policies and guidelines, palliative care is not, integrated into the existing health care system. Other participants responded that palliative care is not well integrated into the undergraduate and postgraduate courses except for limited content in the diploma and a few postgraduate courses. Participants described the challenges for palliative care implementation as follows: many lacked awareness about palliative care; and chronically ill patients other than those with HIV received inadequate care, limited to physical care, some pain management, and psychosocial support rather than comprehensive palliative care. In addition, some participants perceived that palliative care was not within the remit of their service, so families and patients were forced to seek alternative or informal care, including from traditional healers.

**Conclusions:**

Enablers for the improvement of palliative care access in rural and regional health care were identified, including better integration of palliative care into the national health care plan and guidelines; palliative care content in university and college courses; and use of mobile phone technology to facilitate care. And policy makers and responsible stakeholders could consider the palliative care implementation in rural and regional health care settings through a combination of home, community and facility-based models.

**Supplementary Information:**

The online version contains supplementary material available at 10.1186/s12904-023-01283-5.

## Background

Access to palliative care is recognised as an international human right [1]. Relevant global forums and frameworks urge countries to provide access to care, essential medications and goods and services on a non-discriminatory basis to ensure that these rights are respected [1, 2].

Each year, over 56.8 million people worldwide require palliative care, including 31.1 million before and 25.7 million near the end of life [3, 4]. Of 76% of adults in need of palliative care in low- and middle- income countries (LMICs), the majority are from low-income countries [3, 4]. However, less than 5% of those in Africa in need, receive palliative care services, although access has been improving in recent years [5, 6]. The possible reasons could be a lack of morphine, the extreme shortage of health professionals, and palliative care not integrated into the curriculum of health courses and health care system; 45% of African countries had no identified hospice or palliative care [7, 8].

Currently, several palliative care initiatives are underway in Ethiopia. The national health care plan, policies, and guidelines are now integrated palliative care [9–14]. Ethiopia has also trained health care providers [15, 16]. However, palliative care remains primarily donor dependent and focused in urban areas [17–19] despite the majority of the population, more than 78%, residing in rural parts of the country [14, 20]. In this context, rural health services are defined as being provided in the countryside around the villages. For example, the only health service available in this area is a health post where the health extension workers (HEW), also called community health workers (CHW), are responsible for providing illness prevention services. Regional health services are those health services delivered in primary, secondary, and tertiary healthcare services in an administrative state or region of the country [14, 21]. Holistic palliative care refers to the whole spectrum of care involving the physical, social, psychological, cultural and spiritual care of a person [22]. The limited practice of palliative care in Ethiopia is partly linked to low awareness of comprehensive palliative care services among policymakers, health care professionals and community members [17]. Palliative care is also affected by financial and human resources shortages, weak stakeholder collaboration and the absence of a holistic approach [17]. Consequently, for millions of people in Ethiopia, access to palliative care is either limited or absent [14, 17, 18].

There is a lack of data on the available palliative care services and palliative care needs in Ethiopia [17]. A few nonprofit non-governmental organisations [6] such as Hospice Ethiopia, Mary Joy Development Association (MJDA), and Beza for Generation (B4G) provide home-based support [23]. Hospice Ethiopia referred its clients for advanced care to public referral hospitals such as Black Lion hospital (Tikur Anbesa Specialized tertiary hospital), St Paul hospital, and Yekatit hospital in Addis Ababa where palliative care services are delivered [23]. Despite these hospitals have some limited inpatient care [23], none have home-based palliative care programs [17]. In addition, there is limited access to oral morphine and no specific palliative care education is available in the country [6], apart from 1 to 2 trained staff who are working on palliative care in these hospitals [23]. All these services are available in the capital city, Addis Ababa. Rural patients travel to the capital city—in most cases a distance of over 500 km—to access oncologists, pathologists, radiation therapists or palliative medication and care [24, 25]. Therefore, this study aimed to explore the status of palliative care, enabling factors and implementation challenges in Ethiopia’s rural and regional health care settings.

## Methods

### Study design

Enabling factors and implementation challenges for palliative care in rural and regional health care settings were explored using a qualitative case study and a pragmatist worldview approach. The pragmatist worldview provides greater data collection and analysis flexibility [26].

### Study settings

In-depth interviews were conducted with 42 participants drawn from one of the eleven administrative regions in Ethiopia [27]. Ethiopia follows a three-tier health care system: namely, primary health care units (PHCU), secondary care (general hospital) and tertiary care (comprehensive specialized hospital) [14]. The study setting details are reported fully elsewhere [28–30]. The national palliative care guideline described in detail where and how palliative care should be be integrated in each of these levels of health care system [31].

### Study participants and recruitment

The study targeted those professionals who worked in the selected care units in health care settings; regional health representatives, and tertiary health school heads, who had worked in their sectors for at least six months and rural community members who lived in the selected villages for at least six months.

Forty-two participants were purposefully recruited from the conveniently selected units and the settings in the region. Specifically, participants included two policymakers from the Regional Health Bureau, seven pharmacists, five doctors, four health officers (clinical officers) and 17 nurses in leadership roles, including chief nursing officers, who all held leadership roles at all levels of health care institutions. It also included three academic leaders in nursing, medicine, and pharmacy schools in universities, and one the regional health college, as well as three HEWs deployed in rural health posts. The study participants details are described elsewhere [29]. The same framework was used to guide the interview questions for all participants with emphasis on each participant’s speciality. For example, the academic leaders’ questions provided more emphasis on palliative care education, the pharmacists on medication availability, nurses, and doctors more on the implementation of the service. The interviews were conducted in the local language with almost all the participants. However, three of the school leaders and two hospital leaders preferred English so their interviews were conducted in English. Each interview lasted 30–50 min.

Two focus groups were conducted with rural adult community members including farmers (women and men), community and religious leaders. Each focus group comprised eight participants. The focus group questions were designed to identify the strategies used to care for people with a life-limiting illness and the challenges related to caring in the rural areas of the region among the rural community groups. Open-ended questions were posed about the status of palliative care services; the strategies for a sustainable public health approach to the provision of palliative care; and the challenges related to palliative care in all levels of health care settings. The focus groups were conducted in the local language, lasting 45–50 min and were facilitated by the author (AA).

### Data collection

The study was conducted in late 2018. Interview guides were prepared following the four components of public health strategy: policy, education, medication availability and implementation [2]. Questions for the focus groups were adapted from the literature and used to elicit community members’ experience of caring for people with a life-limiting illness [32]. Except with five participants, all the interviews and focus groups were conducted in the local language and facilitated by the author (AA) using an audio recorder. The original recording and translation/transcription were de-identified to maintain confidentiality.

The data were translated from local language to English and back translated into local language and compared, but some words might not have had an exact English translation. The data were translated and transcribed by the author (AA), which could have introduced researcher bias. To reduce this bias, the research team reviewed and questioned the researcher regarding concepts as the results developed.

### Qualitative data analysis

A thematic analysis technique guided the data and evidence synthesis [33, 34]. The thematic analysis utilised the following six steps:- becoming familiar with the data; systematically coding core features from the data; searching and gathering the relevant data and developing potential themes; checking themes for relevance with the codes defining themes; and writing the scholarly report of the final analysis [33]. Finally, the developing themes were synthesised to comprehensively describe palliative care practice and identify barriers and enablers in providing palliative care in rural and regional health care settings. The author (AA) transcribed the audio files verbatim in the language of the interview. Data analysis was supported by the software package NVivo 12.

### Rigor

Rigor of qualitative research operationalised using four criteria including credibility, dependability, confirmability and transferability. The details of rigour are noted elsewhere [28].

## Results

### Theme 1: status of palliative care in rural and regional health care settings

In this theme, the status of palliative care in rural and regional health care settings are described in three sub-themes. These are *palliative care for chronically ill patients*; *disease specific palliative care: the case of HIV patients and holistic palliative care*.

#### Palliative care for chronically ill patients

Participants noted that aspects of palliative care services like physical care, pain management, and psychosocial support were available to chronically ill patients but were largely inadequate or fragmented. A medical doctor participant from a comprehensive specialized hospital noted, “*Oncology unit nurses administer cancer medicines, vomiting and pain management medicines which* [is] *part of palliative care.* [However], *we only focus on pain or pathology of the disease…*” (Participant CTHD). Another medical doctor suggested that they focus on treatment while the nurses provide psychosocial support. “*… palliative care usually focuses on medical treatment … by the physician … and the nurses give the psychological and social support in the clinical ward….*” (Participant 2GHD1).

In the opinion of a clinical nurse leader in an Oncology Unit, holistic palliative care is the responsibility of the clinical ward, not the oncology unit. “*… It is not a ward … we are not delivering comprehensive palliative care in this unit* [oncology]. *The patient is only given chemotherapy and discharged. We do not follow them to the end stage of cancer.*” (Participant CTHWNH1). However, a clinical ward nurse leader in the comprehensive specialized hospital explained that nurses in the clinical wards do not offer holistic palliative care either, as they only focus on physical care. “*The care we* [nurses] *provide in the ward can be feeding, bed bath, and positioning every two hours to prevent bed sores. We do not provide holistic palliative care. We simply offer physical* [care] *…. These are our duties. …*” noted Participant CTHWNH3.

Some participants highlighted that nurses also provide psychological and economic support. “*As nurses … we provide counselling and psychological care …*[and] *economic support for those who have difficulty buying required medications.*” a participant from a comprehensive specialized hospital remarked (CTHWNH1). A rural district nurse further observed, “*…we give psychological support for the patient and their families.*” (Participant 1HEWHP).

Similarly, a comprehensive specialized hospital (CTHND) participant added, “*Some patients do not have money to get treatment. Social workers try to alleviate their* [of the patient] *financial problems … to get them free service in the hospital, or connect them to the funding organisations …*” However, another participant from comprehensive specialized hospital (CTHWNH1) seemed unaware of the existence of social workers. ‘*I am not aware of social workers*.’ The absence of bereavement care for families was also noted: “. *We manage their* [patients] *chronic pain and provide psychological support* [and] *offer* [post-mortem] *care per local customs and cultural practices, but there is no bereavement care”*, reported a general hospital participant (2GHWNH1).

#### Disease-specific palliative care: the case of HIV patients

Other participants commented that palliative care is limited to HIV patients. “*… as a country, … palliative care is implemented for HIV patients as part of comprehensive ART* [Anti-Retroviral Therapy] *care. International NGOs provide the cost of care …*” (Participant 2GHD1). HIV care was not limited to the health care settings as patients needed follow-up at home. “*… palliative care is provided at home for ART patients*” (Participant 2GHD1). Another participant added that “*… they* [professionals] *went to their* [patient’s] *home because there is a concern and attention and a small fund from CDC* [Centres for Disease Control and Prevention] *…*” (Participant 1GHD). Focus group participants noted that HIV patients get support from governmental and non-governmental organisations. “*… HIV program is well supported. But in our district, the main challenge is chronic patients other than HIV… no one looks after them*” (Participant 2RCFG).

The long-term sustainability of palliative care, even for HIV patients, has been questioned, given its over-dependence on donor funds. “*We are dependent on NGOs. For example, we had a local NGO, known as mom-to-mom, in which we implemented standard palliative care for HIV patients as there was a small incentive* [funds] *from NGOs such as I-TECH* [International Training and Education Centre for Health] *and others. But we no longer have this project.*”. (Participant 2HCHO).

#### Holistic palliative care

Participants alluded to the lack of holistic care, irrespective of the disease or hospital setting. A general hospital clinical nurse leader (Participant 1GHWNH1) noted, “*For example, we administer medication, measure vital signs and provide advice for the patient. However, the care we provide is not based on what you said* [holistic palliative care].” A doctor from general hospital also noted, “*We can say …palliative care is only partially implemented here … the professional should offer comprehensive palliative care for the patient, not just pain relief…*” (Participant 1GHD). This participant said that “*I prefer to say that palliative care is more or less not implemented.*” Some participants added that patients diagnosed with incurable illnesses were also given inadequate information on their care options. “*When patients* [are] *discharged from the hospital, they do not get palliative care advice from the doctor or other health professionals…*” (Participant CTHWNH3).

This first theme has discussed the status of palliative care implementation in three different sub-themes. The following two broad themes are constructed to describe the barriers and enablers of palliative care in rural and regional health care settings.

### Theme 2: underlying barriers to palliative care in health care settings

In this theme, four sub-themes emerged: *awareness, leadership and policy-related challenges; education-related challenges; costs of palliative care; and socio-cultural attitudes, norms and other constraints.*

#### Awareness, leadership, and policy-related challenges

Participants said that palliative care had received insufficient attention in policy and should be the concern of national and regional governments. “*Palliative care … as a system …is an ignored service*” (Participant 1GHD). There is no government leadership or “*direction on palliative care. The government should focus on palliative care …*” (Participant 1HEWHP). “*…the federal ministry of health and regional health bureaus should provide all-rounded support, … and should allocate a dedicated budget …*”, added another participant (Participant 2GHD).

Some participants attributed poor palliative care practice in the country to the limited awareness of relevant national health policies and guidelines among educational leaders and health workers at all levels of the health care system. One educational leader noted that: “*I have never heard of a palliative care policy from our government … So, for me, I do not think there is a clear, operational palliative care policy in practice right now …*” (Participant CSH2). Some participants, though they were aware of the National Palliative Care policy and relevant guidelines, felt the documents were only available for a few leaders and were not well distributed to the wards. “*… I have softcopy to use for myself- the palliative care guideline … we did not distribute palliative care guideline to the wards in a hard copy form …*” (Participant CTHND).

Although the regional health bureau had a dedicated portfolio for palliative care, participants said that staff lack the necessary expertise. “*…palliative care is not clear even for those focal persons.*” (Participant CNGOR). Participants added that even the nurses working in the general hospital and primary health care units lacked awareness. “*… if you ask about palliative* [care], *the nurses may ask you what it is?… There is a general lack of awareness among nurses…*” (Participant CRCR).

Another participant also reported that doctors in the comprehensive specialized hospital had low awareness of palliative care: “*… the main challenge is awareness starting from the doctors there is lack of awareness in the whole palliation, psychosocial and spiritual care. They only focus on treatment and pathology and … So, awareness is the problem…*” (Participant CTHD). A general hospital medical doctor concurs, “*… Doctors often have no idea about palliative care. They only know about the aspect of medical care, but I also think* [palliative care] *includes psychological, social and spiritual components and so on …*” (Participant 2GHD1).

#### Education-related challenges

Some participants noted that the medical education curriculum does not cover palliative care: “*We* [school of medicine doctors] *have no power to do that* [integrate palliative care in the curriculum] *as it is done nationally… There are some initiatives, but they are not yet fully harmonised. The national curriculum is revised every five years …*” (Participant CTHD). Other participants clarified that although palliative care is included in the diploma and speciality programs in nursing, teaching about palliative care depends on the level of interest of individual lecturers. Some lecturers provided focus and assessed the students some did not: “*… it is not systematic or organised. The decision to teach palliative or not is a personal decision, left to each instructor…*” (Participant CSH2). In addition, lecturers may not know the subject matter. “*…the instructor may not teach … with the experienced knowledge we are reading and searching what palliative care and details.*” (Participant CRCR).

Others remarked that gaps in the formal curriculum, which could have been filled through in-service training, are also limited. Hence, given the high medical workforce turnover, facilities end up without trained staff. “*… all the doctors are not trained, especially the resident* [postgraduate] *and intern* [graduate]*…*”. (Participant CTHND). *In-service training on palliative care was offered to one or two professionals of the hospital*” (Participant 1GHND*).* “*… previously … there were a few training opportunities available … but there are none recently… palliative care is often not part of the components of in-service training*”, added another participant (Participant CTHND).

Additionally, staff struggled to find health care resources to incorporate the theory they had learned in training into their practice: “*… The trained professional in our ward could not put their knowledge into practice …* [because of], *lack of materials to implement the service. Then we lost the trained staff because of* [hospital staff] *rotation.*” (Participant CTHWNH2). “*… The high staff turnover, especially for doctors, is a major challenge.*” (Participant CTHND).

#### Cost of palliative care

The cost of services impacted on the provision of palliative care. A participant described having “*no sustainable medication supply at all. 90% of medications we use for palliative care … are imported. And this poses a huge challenge for us to provide continuous palliative care to the community, given the scarcity of hard currency in the country.*” (Participant CRHBR). Another mentioned that “*…* [on our hospital] *we continue providing care even though the case is incurable …*[However] *they* [family] *prefer to take the patient so they can die at home because it is expensive to pay for the dead body transport.*” (Participant 2GHWNH1). When the patient and their families knew the diagnosis was incurable, there was a preference for dying at home for cost reasons “*… they* [the community] *do not want to pay for an incurable disease. Just keep being treated for pain that is very demanding* [severe], *so they* [the family and the patient] *will leave the hospital; the patient prefers to die at home.*” (Participant 2GHD1).

Private for-profit options were described: “*in* [name of the region] *we only have one home-based-care: family clinic … it is in* [the name of the capital city] *…* [name of the private clinic] *… have home-based care. It is a for-profit business and unaffordable, but at least it can be a choice for those who can pay*.” (Participant CNGOR). It was suggested that palliative care should have a dedicated budget within the national health care system.

#### Socio-cultural attitudes, norms and other constraints

Some participants mentioned that even when the cost is not an issue, patients and families often sought that the patient die at home. “[There is this patient that] *… the doctors referred to us* [a nearby Health Centre] *for palliative care … A mother aged 85 or 90*, *and we provided care …. However, the family preferred that she dies at home, and requested to take the patient home, then she passed away …*” (Participant 1HOHC).

Community attitudes affect health care professionals’ perspectives. For example: “*… If we do not hold a good attitude …* [at home or out of hospital], *this will get transferred to our workplaces. So, I believe it is not only the hospital work. In general, … Most of the time they* [the community] *are resistant.*” (Participant CTHD). Another participant reported, “*When the patient has pain and other symptoms, the family took the patient to the hospital; if there was no improvement there, they took him to holy water, and if it is beyond that, the family took the patient to the traditional healers.*” (Participant 1RCFG).

It was noted that: “*… the community’s awareness of palliative care is low … the community understand cancer as a killer disease, but they do not know how to manage or what to do, and they search their alternatives …*” (Participant 2PHND). In addition: “*…we do not provide any health education about palliative care to the community.*” (Participant 1GHWNH). Some participants attempted to provide context for the lack of awareness of palliative care among community members: “*… being a poor country, our health education effort focuses on preventable diseases, which account for 70–80% of the disease burden affecting those communities*” (Participant CRHBR). “*… we provide health education to prevent mother-to-child transmission … and communicable diseases such as HIV.”* (Participant 1HEWHP). Similarly, health education programs through the mass media were focused on preventable and curable diseases rather than palliative care: “*… the media are working in prevention health activity, but it* [media] *did not focus on palliative care from the top to the bottom levels. The focus is for the acute and epidemic, not the non-communicable diseases…*” (Participant 2PHND).

### Theme 3: toward a holistic application of palliative care: some enablers

#### Policy framework and service delivery mode-related opportunities

The regional health bureau has a dedicated “*department for curative and rehabilitation services*” with a participant explained that “*palliative care is included in the national health policy … and* [there is] a *countrywide palliative care guideline.*” (Participant CNGOR). Key policy documents, including the national cancer control plan and the health sector transformation plan, also incorporate palliative care. “*…palliative care is included in the* [country’s] *five year* [health service transformation] *plan … the* [government] *aims to integrate palliative care in about 50% of health care facilities in the next two to three years* [2020]” (Participant CRHBR).

This was viewed by some as an ambitious goal, but within reach, as significant improvements had been achieved in other areas of the health system in previous decades. “*The national health system that integrates actions from the regional level up to the* lowest *administrative unit* [Kebele] *has significantly impacted maternal and child health and infectious diseases… This experience comes from the system … We can do the same for palliative care and non-communicable diseases. We have a system that can address both …*” (Participant RHBR). “*We have a golden system. It is connected end to end.*” (Participant CNGOR).

The network of services and the standard of care across the different health care levels, could support the region’s integrated and holistic palliative care. “*… our referral system by itself, which starts from the bottom health post, health centre, primary hospital, general hospital then comprehensive specialized hospital has a* [network] *… we have a standard in every sector with our health education program at the bottom… our community knows about these.*” (Participant CRHBR).

#### Education-related opportunities

Some participants mentioned the opportunities to enhance awareness about palliative care “*… we have universities, colleges …* [in the region] *that can* [provide] *training … we have a lot of nurses who did not get the opportunity to be employed … we can re-train these nurses … and recruit them in palliative care service. Even retired nurses can be brought on board. They can serve as mentors…*” (Participant CNGOR). “*… we have oncologists… They are delivering a good palliation … we have good health professionals in different disciplines … Now the number of sub-specialities is also increasing …*”, added a medical doctor (Participant CTHD). Some participants described the opportunities related to having general practitioners and specialists in the general and primary hospitals: “*… we have a top-level specialist and general practitioners, and we also have nurses who are providing care together with the doctors …*” (Participant 2GHWNH1). Several universities and colleges also train these professionals, which can be harnessed to enhance awareness about palliative care.

There were opportunities to strengthen the palliative care team in the comprehensive specialized hospital discussed: “*… we have a palliative care team … we have … three kinds. One … pain-free, second the thing which emphasised in cancer, we have individuals who focused on palliative care treatment and the third one is we have individuals who start fellowship from American MD* [Medical Doctor] *cancer centre …*” (Participant CTHD).

#### Integrated community-based palliative care

Integrating palliative care into HEW activities is seen as a strategic entry point and an important opportunity to expand palliative care in the community. The country’s health extension program (HEP) was considered as adaptable to providing integrated palliative care services. “*… we have a HEP … which initially had 16 service components and two—non-communicable diseases and mental illness—had been added recently. … So, we already have the system; the thing is … we need to scale up and integrate … those* [palliative care] *services into the system …*” (Participant CRHBR). “*… HEWs already deliver the 18 core services of the country’s HEP to our communities. They move from house to house to deliver these core services. Hence, if they find a chronically ill patient suffering in their home during their routine visits … they can provide palliative care service at the patient’s home, even if they may prefer to die in their home …*” concurred another participant. (Participant 2HCHO). “*HEW are acting as a family with the community. In addition, we have health development army* [also called Women Development Army in Ethiopia, the women from the village served as a primary community engagement platform at the grassroots level] *female volunteers, and farmers and young people who can also support HEWs*” (Participant CRHBR). *We need to* [strengthen] *the health post… We can employ a nurse who can accommodate palliative care at health post. We should strengthen the health post by recruiting professionals who can deliver palliative care …* (Participant CNGOR).

There was further clarification about how the national health insurance program could also be usefully exploited to provide integrated palliative care initiatives in the community. “*… we just started a health insurance system in our region in which 45% of our community is already enrolled. We are planning to reach 80% of the community this year. So, if 80% of the community are registered, they will not have the reason to stay in their home and say ‘I will not go to the hospital’ because of lack of money…*” (Participant CRHBR).

#### Socio-cultural capital and opportunities for home-based care

An NGO representative described the opportunities for home care by involving volunteers, given the respect attached toward the elderly and cultural norms and expectations of providing social support to those with chronic illnesses. “*… our culture* [teaches] *individuals to show kindness toward the sick and seniors. We have volunteers … so excited to provide care for these people…*” (Participant CNGOR). Some participants recounted how the community supported HIV patients and how these community networks can be mobilised in the same way to rally support for terminally ill patients. “*… we have a social group from every community to help for HIV patients … by* [contributing] *cereals and money … The HDAs* [Health Development Army] *mobilise the financial support …*” (Participant 1HEWHP).

“*There are one to five networks* [network of groups, each with a community leader and five community members]. *The group collects some cereals and economic support by collecting money…*” (Participant 1PHHO). Some participants further pointed out socio-cultural practices, such as neighbourhood-based associations in the community, that could be mobilised to help chronically ill patients with no relatives to look after them. “*… we have a good culture; the community can help each other for the chronic patients who have no family … we have ‘Mahber’ or* [local spiritual or political association] *… the neighbour can carry the patient to the hospital or provide support at home…*” (Participant 1HEWHP).

#### Use of mobile technology to facilitate care services

Every household in the district has the phone number of HEWs to assisted in communicating with the health worker. “*Every farmer has the HEWs mobile phone number. If there is an emergency, such as if a pregnant mother needs help, they call us. The farmers use their neighbour’s mobile if they don’t have their own.*” (Participant 2HEWHP).

Participants indicated that this network could be reoriented to provide social support and link patients, health professionals, or NGOs to support home-based palliative care in the community. For example, “w*e already have case managers who facilitates HIV care using mobile phones. The case manager facilitates a support activity for patients and those who require economic support …*” (Participant 2PHHO). “*… this* [the mobile phone] *technology, which is being used for HIV patients, can be used for palliative care. The mobile phone can be used to arrange home visits or* even provide *follow-up over the phone…*” (Participant CTHND).

Mobile phones are also used in the oncology unit to communicate and provide follow-up care for oncology patients. However, an Oncology nurse indicated potential challenges of the technology, including staff workload. “*… previously … we called the patient they* [the family] *get our phone number, and they called us … But now … because of patients’ overload, it is difficult to call the patient …*” (Participant CTHWNH1).

Other participants also argued that using mobile phone technology was a good idea but required more work, such as preparing the palliative care resources required before being used for home-based palliative care. “*… the technology is good, and we have used this technology in mother and childcare. The technology needs further development before being used* for it [palliative care] *in the region …*” (Participant CRHBR).

## Discussion

This study aimed to explore the status of palliative care, enabling factors and implementation challenges in rural and regional health care settings. The World Health Organization (WHO) public health strategy and a thematic analysis approach were used for data analysis and framing study findings [2]. The study showed that despite existing policy frameworks, health care facilities focus on preventable and curable diseases rather than palliative care. As a result, palliative care provided to chronically ill patients was limited to physical care, pain management, and psychosocial support. Comprehensive or holistic care was absent, leaving patients and families to seek alternative care through private clinics, charities, religious organisations, or traditional healers.

Critical systemic barriers to implementing palliative care included: limited national leadership and focus on palliative care; lack of palliative care awareness among political leaders, professionals and community members; cultural norms and attitudes towards chronic diseases; inconsistent pre-service and in-service training; lack of dedicated budget for palliative care.

On the other hand, enabling factors identified included: an integrated referral system and service delivery model; and utilising a reserve health workforce, such as unemployed and retired nurses, and motivated specialists and sub-specialists. Improving the availability and consistency of palliative care education in universities and colleges was considered vital. Private clinics, charities, religious organisations, and volunteer caregivers were identified as potential resources for community-based palliative care. Furthermore, mobile phone technology, ongoing health insurance initiatives, health extension workers and health development army programs were additional factors to improve access to palliative care in regional and rural settings.

The current study’s findings are broadly consistent with previous findings that holistic palliative care was not practiced in health care settings except for some aspects of palliative care, such as pain management, physical care, and psychosocial care. A study conducted in Ethiopia by Kaba et al. [17] reported that actual care practice was only focused on physical care and financial support. Mamo et al. [35] also indicated that the existing care model focused on pain management rather than holistic palliative care.

In contrast, HIV patients receive both home and facility-based palliative care. This findings are consistent with the study findings in Tanzania where the biggest group of palliative care received patients (95%) are HIV infected individuals, the services are provided in hospital, villages and at the patients home [36]. In-service training for staff in ART clinics and home-based palliative care is provided through non-governmental organization (NGO) funding. But current study participants questioned the sustainability of palliative care for HIV patients as it is donor dependent. This finding is consistent with the reviews conducted in low-and middle-income countries, including Africa, indicating that HIV palliative care services are unsustainable because of their donor dependent funding models [6, 36–39].

This study revealed that the regional focal person for palliative care, leaders of academic institutions, and professionals working in all health care settings lacked palliative care awareness. Community awareness about palliative care was equally limited. Despite palliative care being included in the policy documents, professionals such as nurses, educational leaders, and other health care staff were unaware of these documents and even the term ‘palliative care’. However, those in health care leadership positions had the national palliative care guidelines, but they were not shared with staff. These findings are consistent with Kabab et al. [17] and Rhee et al. [39] which found that participants at all levels—i.e. at policy, health facilities and community levels—had poor palliative care awareness. Similarly, they reported that health care professionals were unfamiliar with the Ethiopian national guideline, while patients and caregivers were unaware of the palliative care availability. Hence, it is difficult for patients and their caregivers to use a service they are unaware of and for care providers to provide adequate palliative care.

There are however, several initiatives in the region, and also nationally that could help improve palliative care access in rural and regional areas. For example, Ethiopia’s palliative care service is well integrated into the five-year health care plans and guidelines [14, 17, 31].

The WHO proposes the integration of basic palliative care into primary health care and community health extension workers’ training to improve community access and home-based care [40]. According to a WHO report [40], community health workers could be an enabling factor in providing frequent emotional support for the patient and family and reporting to professionals at community health centres. Similarly, a study conducted by Hannon et al. [41] demonstrated the integration of palliative care in primary healthcare service and community volunteer care in clinics and homes of patients in India. A study conducted in Africa and rural South Africa also reported the vital role of community-based palliative care and the responsibility of community health workers in delivering basic palliative care [39, 42]. This is consistent with the current study findings where participants referred to HEWs, the linked health care system and the strong referral pathway for improving maternal and child health and reducing infectious diseases such as HIV in Ethiopia. Hence, in a similar way, palliative care could be integrated into the country’s primary health care particularly into the activities of community health care workers.

Additionally, palliative care initiatives could be integrated and delivered through existing community-based self-help groups. Earlier studies in Ethiopia reported on the role of ‘iddir’, a self-help neighbourhood association traditionally limited to assisting families during funerals, which started providing basic home-based care and support to HIV patients and families [17, 35, 43]. Similarly, participants in the current study indicated that once the necessary training was received, community volunteers, members of the health development army and other community resources, including ‘Mahber’, a local spiritual or political association of individuals in the community could be enabled to provide some aspects of palliative care. Participants argued that universities and regional health science colleges could organise pre-service and in-service training. At the same time, HEWs could serve as a bridge between these groups and health care staff who provide palliative care services at the tertiary hospital, general hospitals and primary health care units.

This study has a few limitations. Firstly, the study participants and educational institutions were purposefully recruited from one region, so the findings may not be generalisable to other areas in Ethiopia. In addition, due to the COVID-19 pandemic and the recent civil war in Ethiopia, there may be changes in the focus and priorities of the national health policy, and the previously available health services may no longer functional and able to serve the community.

## Conclusion

Palliative care is limited or non-existent in Ethiopia, particularly in rural areas where the majority, more than 78% of the population, live. Current initiatives and research are focused on urban settings and are primarily donor dependent. The study showed that holistic care is largely absent. There remains several barriers including; a lack of awareness about palliative care; patients (other than those with HIV) receiving fragmented palliative care interventions; and palliative care not being integrated into the health system and health curricula. The study also identified enablers that help improve access to comprehensive palliative care through a combination of home, community and facility-based models.

### Electronic supplementary material

Below is the link to the electronic supplementary material.

**Supplementary Material 1: Supplementary file 1**: Interview participants (regional health bureau representative, Medical Director, Chef nursing officer and head nurses)

**Supplementary Material 2: Supplementary file 2**: Community members ((local/national NGO, traditional healers, community and religious leaders)

## Data Availability

The datasets used and/or analysed during the current study are available from the corresponding author on reasonable request.

## List of abbreviations

ART: Anti-Retroviral Therapy
CHW: Community Extension Worker/s
CDC: Centres for Disease Control and Prevention
HEP: Health Extension Program
HEWs: Health Extension Worker/s
HIV/AIDS: Human Immunodeficiency Virus/ Acquired Immunodeficiency Syndrome
I-TECH: International Training and Education Centre for Health
LMIC/s: Low- and middle-income countries
NGO/s: Non-governmental organization/s
WHO: World Health Organization
