# Supplementary material for: Palliative care in Ethiopia’s rural and regional health care settings: a qualitative study of enabling factors and implementation challenges
Source: BMC Palliat Care. 2023 Oct 17;22:156. doi: 10.1186/s12904-023-01283-5 (PMC10580684; doi:10.1186/s12904-023-01283-5)
Supplement: Supplementary file 1 — Supplementary Material 1: Supplementary file 1: Interview participants (regional health bureau representative, Medical Director, Chef nursing officer and head nurses) [file 12904_2023_1283_MOESM1_ESM.docx]

**Supplementary file 1: Interview participants (regional health bureau representative, Medical Director, Chef nursing officer and head nurses)**

**Interview guide**

**An interview framework with the leaders adapted from WHO public health strategy components: policy, education, medication availability and implementation (Callaway, Connor, & Foley, 2018)**

1. Current status of palliative care policy implementation and advocacy plan to implement palliative care

1.1. All national plans and policies include palliative care guideline, are implemented and monitored.

1.2. Palliative care services and health care professionals are entirely financed.

1.2.1. Available of financial and human resources resource to implement palliative care.

1.2.2. Adequate funding for health professionals working in palliative care including reimbursement.

1.3. Opportunities and barriers

2. Essential medicine policy

2.1. Palliative care medicines such as morphine included in the country’s essential medicine list (pharmacy head)

2.2. All doctors have received training on opioid prescribing and are authorised to prescribe (medical directors, regional health bureau representative)

2.3. Availability and affordability of opioids and other Palliative care medicines throughout the country (pharmacy head)

2.4. Pain relieving medications in all forms and doses are available in the country (pharmacy head)

2.5. Distribution, dispensing, and accountability system (pharmacy head)

2.6. Opportunities and barriers

3. Palliative care education in health care professionals

3.1. The policy encouraged palliative care education in curricula and examinations of undergraduate and postgraduate health care students (school heads, regional health bureau representative)

3.2. Palliative care education through in-service training for health care professionals working in each level (regional health bureau representative, medical directors, chief nursing officers, head nurses, school heads)

3.3. Palliative care education for the public such as media and public awareness (regional health bureau representative)

3.4. Opportunities and barriers

4. Integration and implementation of palliative care

4.1. Current palliative care services including home care and inpatient beds in all hospitals, nursing, and elderly homes.

4.2. Current status in supporting family caregivers (regional health bureau representative, health extension workers)

4.3. Technology related service such as a mobile phone.

4.4. Sustainability of care for people diagnosed with life-limiting illnesses.

4.5. Opportunities and barriers

**References**

Callaway, M. V., Connor, S. R., & Foley, K. M. (2018). World Health Organization public health model: a roadmap for palliative care development. *Journal of Pain and Symptom Management, 55*(2), S6-S13.
