# Supplementary material for: Palliative care in Ethiopia’s rural and regional health care settings: a qualitative study of enabling factors and implementation challenges
Source: BMC Palliat Care. 2023 Oct 17;22:156. doi: 10.1186/s12904-023-01283-5 (PMC10580684; doi:10.1186/s12904-023-01283-5)
Supplement: Supplementary file 2 — Supplementary Material 2: Supplementary file 2: Community members ((local/national NGO, traditional healers, community and religious leaders) [file 12904_2023_1283_MOESM2_ESM.docx]

**Supplementary file 2: Community members ((local/national NGO, traditional healers, community and religious leaders)**

**Focus group interview guide**

**Rural community members’ experience of caring for people with a life-limiting illness (McIlfatrick et al., 2014)**

Have you ever had people with incurable illness in this community? Tell me what it was like towards the end of their life?

1. If the patient discharged from the hospital with an incurable illness, what did they do in their home?

2. Who provides care for those patients in their home?

3. What was the role of the family in providing care from terminally ill patients

4. What was the personal preference place of care in the last days of life?

5. What is the source of strength and hope for patient and family on the last day of their lives and to cope with the disease?

6. Do you know any alternative options on how to care for these patients? Can you tell me what they are?

7. If you need information related to caring for life-limiting people, whom do you ask? Where would you look for it?

8. What do you expect from health professionals who care for life-limiting people?

9. Views and opinions of related caring for people who have an incurable illness?

10. If you dream some solutions, what do you think we should have in this community to help care for people with incurable illness?

11. What do you think will make it difficult to in this community to put in place some of these solutions?

12. Some countries have used mobile phone networks to support families in these circumstances. What kind of access do you have to mobile phones?

**Reference**

McIlfatrick, S., Noble, H., McCorry, N. K., Roulston, A., Hasson, F., McLaughlin, D., . . . Kernohan, G. (2014). Exploring public awareness and perceptions of palliative care: a qualitative study. *Palliative Medicine, 28*(3), 273-280.
